# Therapeutical and Pharmaceutical Notes

**Published:** 1900-09

**Authors:** 


### THERAPEUTICAL AND PHARMACEUTICAL NOTES.

Europhen as a Surgical Dressing. Dr. Coleman Nockolds, of Grand Rapids, Mich. (*American Veterinary Review*, March, 1900), states that he prefers europhen to all other surgical dressings as an application to the abdominal wound in ovariectomies. The application of the drug by means of a powder-blower or upon gauze after enucleation of the eyeball also gave excellent results. In eye diseases, such as ophthalmia, simple leucoma, keratitis, and wounds of the conjunctiva and cornea, an ointment of europhen, one drachm to the ounce of lanolin, proved very efficient. For otitis externa and other diseases of the external auditory canal a good application was found to be a powder of equal parts of europhen and boric acid, which was insufflated after cleansing the parts. Europhen proved to be an ideal antiseptic dressing for traumatisms and ulcers, and for wounds following operations on fistulæ and for the removal of tumors. Although a powerful antiseptic, it is non-poisonous, and has but a slight odor. This is of advantage in the treatment of house-pets. It promotes the formation of granulation tissue, allays irritation, and maintains as nearly an antiseptic state as possible in veterinary practice. In skin diseases of the horse and dog, Dr. Nockolds further recommends an application of europhen, one part, in olive-oil, seven parts.

Sterilization of Catgut by Alcohol. Bard (*Gazette des Hopitaux*, November, 1899) sterilizes catgut in alcohol vapor raised to 125° C. This makes the catgut seem stiff, but before use it is soaked in 80 per cent. alcohol. It is very strong, holds its knot, and completely disappears in the tissues in eight days.—*Therapeutic Gazette*.

Healing Powder. A mixture of equal parts of iodoform and calomel will be found to be an excellent antiseptic application for wounds.

Ancient Use of Strychnine.—Strychnine as a therapeutic agent and also as a criminal poison was well known to the ancient Egyptians 1400 B.C.

